# Cost-effectiveness of a fixed-dose combination of solifenacin and oral controlled adsorption system formulation of tamsulosin in men with lower urinary tract symptoms associated with benign prostatic hyperplasia

**DOI:** 10.1186/s12894-015-0031-8

**Published:** 2015-05-09

**Authors:** Jameel Nazir, Lars Heemstra, Anke van Engen, Zalmai Hakimi, Cristina Ivanescu

**Affiliations:** Astellas Pharma Europe Ltd, Chertsey, UK; Quintiles Consulting, Hoofddorp, Netherlands; Astellas Pharma Global Development, Leiden, Netherlands

**Keywords:** Benign prostatic hyperplasia, Cost-effectiveness, Fixed-dose combination, Incremental cost-effectiveness ratio, Lower urinary tract symptoms, Quality adjusted life years, Solifenacin, Tamsulosin, Tolterodine

## Abstract

**Background:**

Storage symptoms, associated with benign prostatic hyperplasia (BPH), often co-exist with voiding symptoms in men with lower urinary tract symptoms (LUTS). Storage symptoms are likely to be most bothersome, and may not be adequately resolved by treatment with α-blocker or antimuscarinic monotherapy. A recent randomised controlled phase 3 trial (NEPTUNE) demonstrated that a fixed-dose combination (FDC) of solifenacin 6 mg plus an oral controlled absorption system (OCAS™) formulation of tamsulosin (TOCAS, 0.4 mg) improved storage symptoms, as well as quality of life, compared with TOCAS alone in men with moderate-to-severe storage symptoms and voiding symptoms. This analysis aimed to assess the cost-effectiveness of a FDC tablet of solifenacin 6 mg plus TOCAS relative to tolterodine plus tamsulosin given concomitantly, from the perspective of the UK National Health Service (NHS).

**Methods:**

A Markov model was developed for men aged ≥45 years with LUTS/BPH who have moderate-to-severe storage symptoms and voiding symptoms. The model calculated cost-effectiveness over an analytical time horizon of 1 year and estimated total treatment costs, quality adjusted life years (QALYs) and incremental cost-effectiveness ratio.

**Results:**

The FDC tablet of solifenacin 6 mg plus TOCAS was associated with lower total annual costs (£860 versus £959) and increased QALYs (0.839 versus 0.836), and was therefore dominant compared with tolterodine plus tamsulosin. Time horizon, discontinuation or withdrawal rates, drug cost and utility values were the main drivers of cost-effectiveness. The probability that the FDC tablet of solifenacin 6 mg plus TOCAS is cost-effective was 100% versus tolterodine plus tamsulosin, at a willingness-to-pay threshold of £20,000/QALY gained.

**Conclusions:**

The FDC tablet of solifenacin 6 mg plus TOCAS provides important clinical benefits and is a cost-effective treatment strategy in the UK NHS compared with tolterodine plus tamsulosin for men with both storage and voiding LUTS/BPH.

**Electronic supplementary material:**

The online version of this article (doi:10.1186/s12894-015-0031-8) contains supplementary material, which is available to authorized users.

## Background

The term ‘lower urinary tract symptoms’ (LUTS) is used to describe a condition that encompasses storage, voiding and post-micturition symptoms [1,2]. The aetiology of LUTS can be multifactorial [2,3], but BPH is a common cause in men. Storage symptoms (e.g. urgency, frequency, urgency incontinence and nocturia) and voiding symptoms (e.g. weak or intermittent urinary stream, straining, hesitancy, terminal dribbling and incomplete emptying) are common and frequently co-exist in men with LUTS [4,5]. Storage symptoms represent the most troublesome LUTS, reported in up to 42% of men aged ≥75 years [4]. Storage symptoms are also reported to be the most bothersome LUTS [6].

Overall, the recommended treatment options for men with moderate-to-severe LUTS include α-blockers, 5α-reductase inhibitors (in those with a large prostate, 30 g or 40 mL) and antimuscarinic (in those with predominant storage symptoms) [2,4,7]. In addition, α-blocker plus antimuscarinic combination treatment should be considered for patients not adequately responding to monotherapy of either drug [2,4]. However, the majority of men with moderate-to-severe LUTS associated with BPH receive α-blocker monotherapy only [8], whilst less than 25% are reported to receive an antimuscarinic [8,9]. Additionally, α-blocker monotherapy is reported to improve voiding and storage symptoms in men with LUTS/BPH [10,11]. However, storage symptoms may persist in some men after receiving α-blocker monotherapy, epitomised by data from Lee et al. that reported only 35% of men with storage symptoms were sufficiently controlled by this treatment strategy [12].

Several trials have demonstrated that α-blocker plus antimuscarinic combination treatment is more effective than α-blocker monotherapy for men with moderate-to-severe LUTS and documented storage symptoms [13-19]. The most recent phase 3 trial (NEPTUNE), which included 1,334 men with LUTS/BPH who had moderate-to-severe storage symptoms and voiding symptoms, showed that solifenacin 6 mg plus an oral controlled absorption system (OCAS™) formulation of tamsulosin (TOCAS) improved storage symptoms and quality of life compared with TOCAS alone [18]. The combination treatment was also well tolerated and exhibited an adverse event profile similar to that reported for the individual monotherapies. A once-daily, FDC tablet of solifenacin 6 mg plus TOCAS 0.4 mg aimed at treating both storage and voiding symptoms in men with LUTS/BPH is licensed and available in several countries, including the UK [20].

The aim of this study was to perform a cost-effectiveness analysis for a once-daily FDC tablet of solifenacin 6 mg plus TOCAS (0.4 mg) versus daily tolterodine extended release (ER, 4 mg) plus tamsulosin (0.4 mg) given concomitantly, in men with LUTS/BPH who have moderate-to-severe storage symptoms and voiding symptoms within the UK healthcare setting.

## Methods

### Model overview

A Markov model was developed to compare the cost-effectiveness of a FDC tablet of solifenacin 6 mg plus TOCAS versus tolterodine plus tamsulosin given concomitantly over an analytical time horizon of 1 year from the perspective of the UK NHS (Table 1). A 4-week cycle period was employed, the minimum time interval used to detect treatment differences in LUTS clinical trials. Inputs for effectiveness data, costs and utilities were extracted from published sources and interviews with clinical experts, as described in detail below. The model provided outcome estimates for total treatment costs, QALYs and incremental cost-effectiveness ratio (ICER). The model was programmed in Microsoft Excel. No ethics or consent were required for this study.

**Table 1:** **Cost effectiveness model overview**

**Aspect**	**Details**
Analytical method	Markov state transition model incorporating a decision tree
Software used	Microsoft Excel 2010
Model perspective	UK NHS
Time horizon	1 year
Cycle length	4 weeks
Patient population	Men with LUTS/BPH who have moderate-to-severe storage symptoms (≥8 micturitions/day and ≥2 urgency episodes/day*) and voiding symptoms
Treatments	Once-daily FDC tablet of solifenacin 6 mg plus TOCAS 0.4 mg
	Tolterodine ER 4 mg plus tamsulosin 0.4 mg daily, given concomitantly
Outcomes	Total treatment costs
	Quality adjusted life years
	Incremental cost-effectiveness ratio

*Patient Perception of Intensity of Urgency Scale, grade 3 or 4.

BPH, benign prostatic hyperplasia; ER, extended release; FDC, fixed-dose combination; LUTS, lower urinary tract symptoms; TOCAS, oral controlled absorption system (OCAS™) formulation of tamsulosin.

### Patients

The model considered men with LUTS/BPH who had moderate-to-severe storage symptoms and voiding symptoms, defined by ≥8 micturitions/day and ≥2 urgency episodes/day (Patient Perception of Intensity of Urgency Scale [PPIUS] grade 3 or 4 [21]).

### Treatment pathway

Men entering the model were treated with daily regimens of FDC tablet of solifenacin 6 mg plus TOCAS (0.4 mg) or tolterodine ER (4 mg) plus tamsulosin (0.4 mg) given concomitantly. After a first treatment period of 4 weeks, men could have experienced a treatment response or no response, based on changes in total urgency and frequency score (TUFS) of ≥6 or <6 points, respectively, estimated as the minimally important difference [22]. TUFS is a validated instrument that captures storage symptoms (urgency and frequency) in a single parameter; TUFS is calculated as the sum of the PPIUS scores (grading of 0 to 4 for each void) recorded in a patient’s micturition diary divided by the number of days recorded in the diary [18].

**Figure 1:**
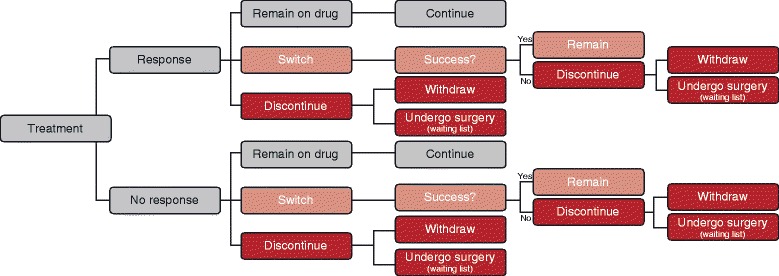
Patient flow diagram.

Patients, either with or without a response may remain on drug or discontinue the treatment (cope with symptoms or wait for surgery to alleviate symptoms) at any model cycle (Figure 1). After 12 weeks (three cycles), patients were permitted to switch to a different combination regimen. After the first 12 weeks, the treatment effect was assumed to be stable (no improvement or deterioration in TUFS).

### Outcomes

The model estimated the following outcomes: total treatment costs; QALYs gained; and ICER. All results are expressed on a per patient basis.

### Model input parameters

#### Assumptions

Several assumptions were made in the model to reflect clinical practice (Additional file 1: Table S1). Input was sought from a group of five clinical experts (two general practitioners [GPs] and three urologists) from the UK, to validate the model input parameters for where data were limited and to fill any data gaps (i.e. surgery, persistence and treatment switching).

### Transition probabilities

Transition probabilities for FDC tablet of solifenacin 6 mg plus TOCAS during the first three cycles were derived from the NEPTUNE study (Table 2) [18]. Tolterodine plus tamsulosin was assumed to have the same treatment effect as FDC tablet of solifenacin 6 mg plus TOCAS (Additional file 1: Table S1).

**Table 2:** **Transition probabilities for the first three cycles** [18]

**Model cycle**	**From response to**		**From no response to**	
	**Response**	**No response**	**Response**	**No response**
1	0.000	0.000	0.539	0.461
2	0.843	0.157	0.278	0.722
3	0.878	0.122	0.248	0.752

### Persistence, switching and surgery

Patients may have discontinued treatment at the end of each cycle due to adverse events or perceptions of efficacy (e.g. satisfaction or dissatisfaction with efficacy; an assumption was made that patients may discontinue treatment despite a positive clinical benefit and/or no tolerability issues). Discontinuation and switching rates were derived from a large observational study of UK primary care between January 2004 and September 2011 (The Health Improvement Network [THIN] database). The analysis included men aged ≥45 years who had an initial diagnosis, symptoms or therapies indicative of LUTS/BPH, and found that over a median follow-up of 2 years, 43.0% and 59.8% of men discontinued solifenacin and tolterodine, respectively [8] (Table 3). In addition, switching rates of 15.3% and 23.3% for solifenacin and tolterodine were reported from the THIN database.

**Table 3:** **Discontinuation and switching rates for responders and non-responders [8 and Interviews with clinical experts]**

	**Responders**		**Non-responders**	
	**2 year**	**4-weekly rate**	**2 year**	**4-weekly rate**
Discontinuation rates				
FDC tablet solifenacin 6 mg + TOCAS	43.0%	0.023	53.0%	0.031
Tolterodine + tamsulosin	59.8%	0.037	69.8%	0.049
Switching rates				
FDC tablet solifenacin 6 mg + TOCAS	15.3%	0.007	15.3%	0.007
Tolterodine + tamsulosin	23.3%	0.011	23.3%	0.011

FDC, fixed-dose combination; TOCAS, oral controlled absorption system (OCAS™) formulation of tamsulosin.

To accommodate the possibility of surgical treatment in the model, it was assumed that 50% of the patients who discontinued the treatment would be eligible for a surgical procedure within 6 months and, consequently, would discontinue drug treatment [4]. Transurethral resection of the prostate (TURP) was chosen as the surgical procedure because it is the current surgical standard procedure for men with LUTS secondary to BPH [2]. The 6-month probability of surgery for TURP was converted into a 1-month probability, assuming that 11% of patients received surgery every month.

### Quality of life

Utility values were derived from EQ-5D data collected in the NEPTUNE study (Table 4) using the UK tariffs. Withdrawal and discontinuation were assumed to have the same utility as the baseline. The average of the response and non-response health state was used to calculate the second-line treatment utility weight, as specific efficacy data were not available. The utility for the post-surgery health state was derived by combining disutilities from DiSantostefano et al. [23] and the response utility value from the NEPTUNE study [18] with the probabilities of improvement, no improvement and adverse events after surgery [23]. Mapping algorithms were also used to derive utilities from a disease-specific instrument overactive bladder questionnaire (OAB-5D) as part of the sensitivity analysis [Astellas, data on file].

**Table 4:** **Utility weights per health state**

**Health state**	**Derivation**	**Utility weight**
Baseline	Based on average utilities of patients at baseline	0.848
Response	Value at Week 12	0.887
No response	Value at Week 12	0.870
Second-line treatment	Average of response and no response health states	0.879
Withdrawal	Assumed to be equal to the baseline utility	0.848
Discontinuation	Assumed to be equal to the baseline utility	0.848
Post-surgery	Derived by combining response utility value from NEPTUNE study with disutilities from [23], weighted by probability of improvement, no improvement and adverse events after surgery [23]	0.839
Death	Lowest utility possible	0.000

### Mortality

The mean age of the men in the model was determined to be 66 years – consistent with the mean age (65.4 years) of the randomised men in the NEPTUNE study [18]. The annual mortality probability for the population was assumed to be the same as that of men aged 66 years from the UK general population (2008–2010) [24].

### Costs and resource utilisation

Costs in the model accounted for the resource utilisation associated with all primary care and hospital-based treatments. Costs were considered over the whole model period and were based on the assignment of fixed costs to health states and transitions between health states (Table 5). Direct costs included drug acquisition costs, healthcare professional visits, surgery, hospitalisation time, and treatment of adverse events. All costs were based on 2013 prices and expressed in British pounds (£). Where 2013 unit costs were not available, costs were adapted to 2013 values using the consumer price index. Costs and outcomes were discounted at 3.5% per annum, as recommended by NICE [25].

**Table 5:** **Treatment costs**

**Treatment**	**Description**	**Price (£)**	**Source**
FDC tablet solifenacin 6 mg + TOCAS 0.4 mg	One tablet per day	0.92*^‡^	BNF [26]
Tolterodine 4 mg + tamsulosin 0.4 mg	One tablet + one capsule per day	1.10 (=0.92 + 0.18)^‡^	BNF [26]
GP visit	Per clinic consultation lasting 17.2 minutes, excl. direct care costs, incl. qualification costs	230.0	PSSRU [41]
Surgery	Prostate transurethral resection procedure 80% without CC, 20% with major CC	2,643.4^§^	NHS [42]; Antoñanzas et al. [43]

*Price parity with solifenacin 5 mg (£0.92/day); Prescription charge excluded from UK analysis. ^‡^Price per day. ^§^Price calculated according to Antoñanzas et al. [43]: 20% of LB25F plus 80% of LB25D.

CC, complications and comorbidities; FDC, fixed-dose combination; GP, general practitioner; TOCAS, oral controlled absorption system (OCAS™) formulation of tamsulosin.

Drug acquisition costs were obtained from the British National Formulary [26] taking into account the daily dose (Table 5). Patients were assumed to have regular GP/urologist follow-up visits every 6 months; additional visits were planned for the switching or discontinuation of treatment. All surgical procedures were assumed to be TURP based on advice from interviews with clinical experts.

### Sensitivity analyses

Deterministic and probabilistic sensitivity analyses were performed to determine the influence of uncertainty on the final results. A standard deterministic univariate sensitivity analysis was performed on all model parameters, varying each parameter through a plausible range whilst holding other parameters fixed and assessing the effect on the overall outcomes and the ICER. Results of these analyses are presented using a tornado diagram. A tornado diagram visualises and orders the model parameters from those that have the highest impact on incremental model results to parameters that have the lowest impact on incremental outcomes.

In the probabilistic sensitivity analyses (PSA), parameter estimates were varied within their uncertainty distributions that best reflect the nature of each specific parameter. Aligned with standard methods [27], gamma distributions were selected for costs, beta distributions for probabilities and utility values, and a Dirichlet distribution was used for transitions in the first 12 weeks of the model. Monte Carlo simulations (n = 1,000) were performed using randomly selected values from the probability distribution assigned to each parameter. The results of the PSA are presented in the form of a graph displaying the results of the 1,000 simulations on the cost-effectiveness plane.

Several scenarios analyses were performed using alternative discontinuation rates, time horizons and utility values. The discontinuation scenario analysis utilised an alternative discontinuation rate for tolterodine, which was based on a report of prescriptions for antimuscarinic therapies in the UK [28]. This report indicated that discontinuation for tolterodine versus solifenacin had a relative ratio of 1:10. Consequently, an alternative 4-weekly discontinuation rate of 0.026 for tolterodine (46.8% over 2 years) was applied to the model (compared with 0.037 in the base case model). In the time horizon scenario, the cost-effectiveness for FDC tablet of solifenacin plus TOCAS versus tolterodine plus tamsulosin was calculated over four time horizons of 1, 3, 5 and 10 years. Additionally, the utility values for each health state were replaced with OAB-5D-derived utility values (Additional file 1: Table S2).

## Results

### Base case results

A higher proportion of men treated with the FDC tablet of solifenacin 6 mg plus TOCAS were still on their original treatment compared with tolterodine plus tamsulosin at Week 12 (92.0% versus 87.6%, respectively) and at 1 year (65.0% versus 50.5%, respectively), and a higher proportion of men had a response (56.9% versus 54.4% at 12 weeks, and 41.5% versus 32.8% at 1 year). Additionally, the proportion of men in the post-surgery health state at 1 year was smaller for the FDC tablet of solifenacin 6 mg plus TOCAS (6.9%) compared with tolterodine plus tamsulosin (10.2%) (Table 6).

**Table 6:** **Base case results: Distribution of patients across the health states**

**Base case scenario**	**FDC tablet solifenacin 6 mg + TOCAS**	**Tolterodine + tamsulosin**
Patient in HS1	41.46%	32.76%
Patient in HS2	23.56%	17.76%
Patient on second-line treatment	4.30%	6.50%
Patient withdrawn	14.60%	20.75%
Patient who discontinued treatment	7.71%	10.57%
Patient in post-surgery	6.89%	10.18%
Dead patient	1.48%	1.48%

FDC, fixed-dose combination; HS1, Response health state; HS2, No response health state; TOCAS, oral controlled absorption system (OCAS™) formulation of tamsulosin.

After 1 year, the FDC tablet of solifenacin 6 mg plus TOCAS was associated with lower annual per patient total costs (£860 versus £959, respectively) and increased QALYs (0.839 versus 0.836) compared with tolterodine plus tamsulosin (Table 7). The FDC tablet of solifenacin 6 mg plus TOCAS was therefore dominant (i.e. more effective and less costly) compared with tolterodine plus tamsulosin (Table 7).

**Table 7:** **Base case results: cost-effectiveness**

	**FDC tablet solifenacin 6 mg + TOCAS**	**Tolterodine + tamsulosin**
Total costs* (£)	860	959
Difference	–	−99
QALYs*	0.840	0.836
Difference	–	0.002
ICER*^‡^	–	Dominates (−£40,469)

*Per patient at 1 year.

^‡^FDC tablet solifenacin 6 mg + TOCAS versus tolterodine + tamsulosin.

FDC, fixed-dose combination; ICER, incremental cost effectiveness ratio; QALY, quality of life adjusted years; TOCAS, oral controlled absorption system (OCAS™) formulation of tamsulosin.

### Sensitivity analyses

**Figure 2:**
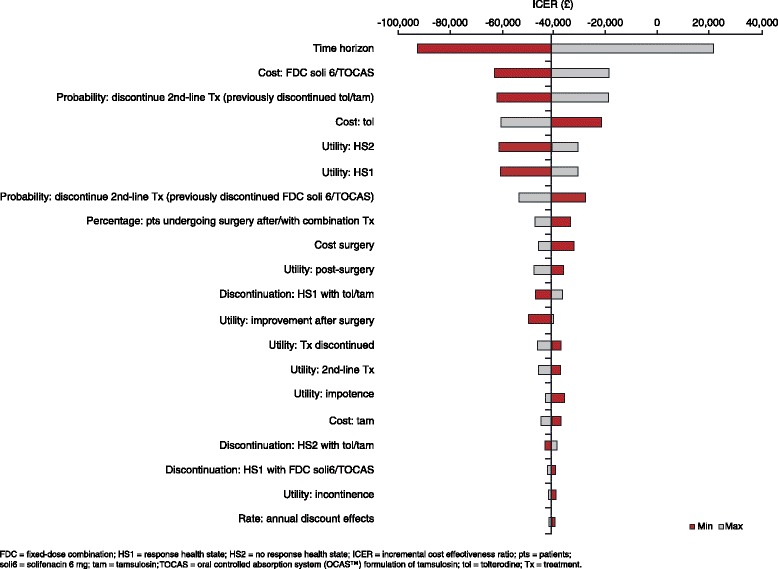
Cost-effectiveness Tornado diagram: FDC tablet solifenacin 6 mg + TOCAS versus tolterodine + tamsulosin.

The univariate analysis showed that the model was most sensitive to time horizon, discontinuation/withdrawal rates, drug cost and EQ-5D-derived utility values (Figure 2). The FDC tablet of solifenacin 6 mg plus TOCAS remained dominant (i.e. costs less and generates more QALYs) or was cost-effective (i.e. ICER below the £20,000 threshold) compared with tolterodine plus tamsulosin in all parameters except time horizon.

**Figure 3:**
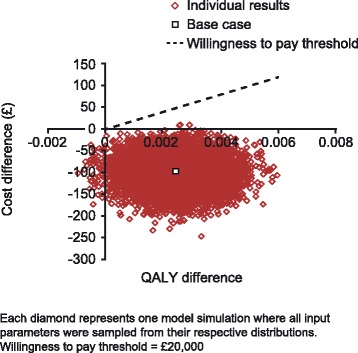
Cost-effectiveness scatter plot: FDC tablet solifenacin 6 mg + TOCAS versus tolterodine + tamsulosin.

The PSA showed that the annual per patient mean incremental cost was –£99 (standard deviation [SD], £33) and the incremental QALYs was 0.0019 (SD, 0.0002), showing that the FDC tablet of solifenacin 6 mg plus TOCAS remained dominant compared with tolterodine plus tamsulosin (mean ICER, −£51,941; Figure 3). At a willingness-to-pay (WTP) threshold of £20,000 per QALY gained, the probability that the FDC tablet of solifenacin 6 mg plus TOCAS is cost-effective was 100% versus tolterodine plus tamsulosin.

### Scenario analyses

An analysis that used an alternative discontinuation rate for tolterodine plus tamsulosin, as determined by Wagg et al. [28], indicated that the FDC tablet of solifenacin 6 mg plus TOCAS remained dominant compared with tolterodine plus tamsulosin (Table 8). Similarly, a scenario analysis performed using OAB-5D-derived utilities showed that the FDC tablet of solifenacin 6 mg plus TOCAS remained dominant compared with tolterodine plus tamsulosin with larger incremental QALYs (0.0005) after 1 year (Table 8).

**Table 8:** **Scenario analyses: cost-effectiveness**

	**Discontinuation analysis**		**OAB-5D analysis**	
	**FDC tablet solifenacin 6 mg + TOCAS**	**Tolterodine + tamsulosin**	**FDC tablet solifenacin 6 mg + TOCAS**	**Tolterodine + tamsulosin**
Total costs* (£)	860	942	860	959
Difference	–	−82	–	−99
QALYs*	0.839	0.838	0.835	0.831
Difference	–	0.0006	–	0.004
ICER*^‡^	–	Dominates (−£133,473)	–	Dominates (−£26,143)

*Per patient at 1 year.

^‡^FDC tablet solifenacin 6 mg + TOCAS versus tolterodine + tamsulosin.

FDC, fixed-dose combination; ICER, incremental cost effectiveness ratio; QALY, quality of life adjusted years; TOCAS, oral controlled absorption system (OCAS™) formulation of tamsulosin.

A time horizon analysis up to 5 years showed that the incremental difference in QALYs and total annual costs for the FDC tablet of solifenacin 6 mg plus TOCAS compared with tolterodine plus tamsulosin were proportionally smaller with increasing time (Table 9).

**Table 9:** **Scenario analysis: time horizon**

	**FDC tablet solifenacin 6 mg + TOCAS vs tolterodine + tamsulosin**			
	**1 year**	**3 years**	**5 years**	**10 years**
Cost difference* (£)	−99	26	223	404
QALY difference*	0.002	0.011	0.017	0.018
ICER*^‡^	Dominant (−£40,469)	£2,351	£13,531	£22,224

*Per patient.

^‡^FDC tablet solifenacin 6 mg + TOCAS versus tolterodine + tamsulosin.

FDC, fixed-dose combination; ICER, incremental cost effectiveness ratio; QALY, quality of life adjusted years; TOCAS, oral controlled absorption system (OCAS™) formulation of tamsulosin.

## Discussion

There are a few reports of cost-effectiveness of drug treatment in LUTS, but this study represents the first cost-effectiveness analysis of a FDC tablet of solifenacin 6 mg plus TOCAS. Overall, the results of this analysis indicate that the FDC tablet of solifenacin 6 mg plus TOCAS is a cost-effective treatment option for men with LUTS/BPH who have moderate-to-severe storage symptoms and voiding symptoms. The base-case analysis showed that the FDC tablet of solifenacin 6 mg plus TOCAS is dominant (i.e. was associated with improved patient outcomes and lower costs) versus tolterodine plus tamsulosin over a 1-year time horizon.

The robustness of our cost-effectiveness model is demonstrated through the results of the univariate and probabilistic sensitivity analyses, as well as the scenario analyses. The univariate analysis showed that several of the main drivers for superior cost-effectiveness of FDC solifenacin 6 mg plus TOCAS versus tolterodine plus tamsulosin were inputs related to treatment persistence. Data from several areas of medicine describe that adherence/persistence with medication is a key driver of cost-effectiveness [29-32]. Two reports of real-world clinical practice data in the UK indicate improved persistence for solifenacin versus tolterodine in men with LUTS/BPH or overactive bladder (OAB). The THIN database reported that a lower proportion of men with LUTS/BPH discontinued and switched treatment (43% and 15%, respectively) compared with tolterodine (60% and 23%, respectively) over a median follow-up of 2 years [8]. In addition, 35% of patients with OAB were still receiving solifenacin after 12 months compared with 28% for tolterodine ER [28]. Further analyses should be conducted to confirm these observations, and various factors are likely to impact persistence. For example, solifenacin is reported to provide an improved efficacy (urgency and micturitions) and tolerability (dry mouth) profile compared with tolterodine [33]. Subsequently, this may contribute to the increased persistence with solifenacin, resulting in fewer patients discontinuing medication, reduced switching and/or surgery costs, and improved quality of life. This is supported by the slightly better outcomes, QALY gains and lower overall costs, reported in our analysis.

Time and quality of life utility values were also key drivers of cost-effectiveness in our model. The time horizon analysis showed that the FDC tablet of solifenacin 6 mg plus TOCAS remained dominant at the 3-year time horizon and within a generally acceptable range of cost-effectiveness for up to 10 years. The robustness of our model was also exemplified by data showing that the FDC tablet of solifenacin 6 mg plus TOCAS remained dominant when utilities were derived from both generic (EQ-5D) and disease-specific (OAB-5D) instruments. These data are underscored by the NEPTUNE study quality of life data, which reported significant improvements in International Prostate Symptom Score (IPSS) quality of life and OAB-q health-related quality of life total and coping, sleep, concern, and social subscores with FDC tablet of solifenacin 6 mg plus TOCAS compared with TOCAS monotherapy [18].

Data suggest that first-line α-blocker monotherapy may not adequately control symptoms in men with LUTS associated with BPH [12]. As such, current guidelines recommend α-blocker plus antimuscarinic combination as a treatment option for men with moderate-to-severe storage symptoms if symptom relief has been insufficient with the monotherapy of either drug [2,4]. This recommendation is supported by the results of several large randomised trials that have reported improved symptoms and quality of life with combination/add-on therapy compared with α-blocker monotherapy in patients with LUTS [14,15,17,18,34,35]. However, data from a large population-based study, THIN, indicate that α-blocker plus antimuscarinic combination treatment is used in only a small proportion (~15%) of patients with LUTS/BPH who have both storage and voiding symptoms [8]. Overall, these data suggest that there may be an unmet need in this patient population, based on the low use of combination therapy in clinical practice despite its proven effectiveness in men with LUTS/BPH who have both storage and voiding symptoms.

This *de novo* model may have some limitations. First, due to lack of published data, some assumptions were made using expert opinion only, including resource use and the proportion of patients going on to have surgery. Other key assumptions were required, for example due to the absence of persistence data on FDCs or free combinations in LUTS, and due to there being no head-to-head studies for the combinations assessed in the present study. Additionally, the primary trials for the combination therapies evaluated in our analysis had some notable differences in the patient populations and outcome measures that prohibit an indirect treatment comparison. Patients in these trials had IPSS ≥12 or 13, ≥2 or 3 urgency episodes/24 hours and ≥8 micturitions/24 hours. In contrast to NEPTUNE, TIMES had an inclusion criterion for overactive bladder symptoms but not one for voiding symptoms. In addition, the primary efficacy endpoint in TIMES was the Perception of Treatment benefit question [36] and the secondary endpoints included bladder diary variables, and change in episodes/24 hours of urgency urinary incontinence, urgency, total micturitions and night-time micturitions. In NEPTUNE, the co-primary endpoints were total IPSS and TUFS.

Second, although the model included tamsulosin, solifenacin and tolterodine, which are commonly prescribed for men with LUTS [8], other common α-blockers (e.g. alfuzosin) and antimuscarinics (e.g. oxybutynin) were not considered in our model. Additionally, although men with LUTS may receive α-blocker or antimuscarinic monotherapy, our model was restricted to evaluation of combination treatment only. Therefore, future models will be required to compare the cost-effectiveness of monotherapy versus combination therapy and to compare other feasible combination therapies.

Third, the model allowed treatment to be discontinued at any cycle (i.e. every 4 weeks), but switching of treatment was not allowed until 12 weeks; this cut-off is consistent with the assessment point of several recent large randomised clinical trials in LUTS [14,18]. However, it is feasible that switching could occur before Week 12 in clinical practice for tolerability, efficacy or other reasons.

Fourth, the switching and discontinuation rates applied to the model were based on data for antimuscarinics only. This was because, to our knowledge, there are no published data reporting the long-term (e.g. ≥1 year) persistence of α-blocker plus antimuscarinic combination therapy in men with LUTS/BPH.

There are a limited number of published cost-effectiveness analyses for combination treatment with α-blockers plus 5α-reductase inhibitors for men with BPH [23,37-39], but only one published report of α-blockers plus antimuscarinic combination therapy in men with LUTS [40]. The cost-effectiveness analyses in BPH found that combination treatment appears to be largely more cost effective than monotherapy [37-39]. Similarly, a secondary analysis of the TIMES study showed that tolterodine plus tamsulosin appears to be more cost-effective compared with tolterodine monotherapy (dominant) or tamsulosin monotherapy (ICER, 10,381/QALY) in patients with LUTS over a 1-year time horizon [40]. Consistent with our analysis, the higher drug acquisition costs of tolterodine plus tamsulosin were offset by the improved efficacy (postponement of surgery) and quality of life benefits with combination treatment. However, there were some differences between these two cost-effectiveness analyses of combination treatment in LUTS, including that the TIMES model did not incorporate resources associated with GP visits.

## Conclusion

The FDC tablet of solifenacin 6 mg plus TOCAS has been demonstrated to significantly improve storage symptoms and quality of life compared with TOCAS alone in men with moderate-to-severe storage symptoms and voiding symptoms [18]. This analysis shows that the FDC tablet of solifenacin 6 mg plus TOCAS is also a cost-effective treatment strategy compared with tolterodine plus tamsulosin for this population of men, from the perspective of the UK NHS. Overall, these data suggest that the introduction of a FDC tablet of solifenacin 6 mg plus TOCAS offers clinical and financial benefits for management of men with LUTS/BPH who have both storage and voiding symptoms.

## Additional file

Additional file 1:
**Table S1.** Key model assumptions [18,23,28,44-46]. **Table S2.** Alternative utility weights per health state.

## Abbreviations

BNF: British National Formulary
BPH: Benign prostatic hyperplasia
EQ-5D: EuroQol 5-Dimensions
ER: Extended release
FDC: Fixed-dose combination
GP: General practitioner
ICER: Incremental cost-effectiveness ratio
IPSS: International Prostate Symptom Score
LUTS: Lower urinary tract symptoms
NHS: National Health Service
NICE: National Institute for Health and Clinical Excellence
OAB: Overactive bladder
OAB-q: Overactive bladder questionnaire
OCAS™: Oral controlled absorption system
PPIUS: Patient Perception of Intensity of Urgency Scale
PSA: Probabilistic sensitivity analysis
QALYs: Quality-adjusted life years
SD: Standard deviation
THIN: The Health Improvement Network
TOCAS: Oral controlled absorption system (OCAS™) formulation of tamsulosin
TUFS: Total urgency and frequency score
TURP: Transurethral resection of the prostate
UK: United Kingdom
WTP: Willingness to pay
