# Supplementary material for: Cost-effectiveness of a fixed-dose combination of solifenacin and oral controlled adsorption system formulation of tamsulosin in men with lower urinary tract symptoms associated with benign prostatic hyperplasia
Source: BMC Urol. 2015 May 9;15:41. doi: 10.1186/s12894-015-0031-8 (PMC4456721; doi:10.1186/s12894-015-0031-8)
Supplement: Additional file 1: — Table S1. Key model assumptions [18,23,28,44-46]. Table S2. Alternative utility weights per health state. [file 12894_2015_31_MOESM1_ESM.docx]

## Supplementary Table 1. Key model assumptions

| Assumption | Justification | Source |
| --- | --- | --- |
| Patients may discontinue treatment even if the treatment is successful and there are no adverse events | Persistence with long-term medication in chronic diseases is typically low | Wagg, et al. [28] |
| 50% of the patients who discontinue the treatment undergo surgery within 6 months | Information provided by UK experts based on their clinical practice/experience | UK clinical expert panel (2012) |
| Patients see their GP once in 6 months (regular visit) | Patients receiving drug treatment for moderate-to-severe LUTS should be reviewed after 4–6 weeks and then every 6–12 months | NICE [44] |
| Surgery only refers to TURP | Other interventions such as minimally invasive surgical treatment are relatively new and don’t provide long-term safety and efficacy data | N/A |
| The short-term success rate of surgery is 70% | Information provided by UK experts based on their clinical practice/experience | UK clinical expert panel (2012) |
| The success rate of surgery is independent of the previous treatment | Information provided by UK experts based on their clinical practice/experience | UK clinical expert panel (2012) |
| Second-line treatment differs between the two interventions | Assumption based on practicability and using the drugs already incorporated into the model: after FDC tablet solifenacin 6 mg plus TOCAS = tolterodine plus tamsulosin; after tolterodine plus tamsulosin = solifenacin plus tamsulosin | N/A |
| 50% of patients who discontinue first-line treatment switch to second-line treatment | Assumption, no additional data identified | N/A |
| Utility of being in the post-surgery health state | Weighted average of the probability of each adverse event and the associated disutility | DiSantostefanos, et al. [23] |
| Utility of treatment ‘Withdrawal’ and ‘Discontinued’ | Assumed to be equal to baseline utility | N/A |
| Utility of being on second-line treatment | Assumed this to be equal to the average of utility of the HS1 and HS2 as no efficacy data are available to distinguish between health states | N/A |
| FDC tablet solifenacin 6 mg plus TOCAS and tolterodine plus tamsulosin had the same treatment effect and transition probabilities | The different patient populations and clinical trial designs for phase III studies of solifenacin plus TOCAS (e.g. NEPTUNE) and tolterodine plus tamsulosin (e.g. TIMES) prohibit an indirect treatment comparison | N/A |
| After the first 3 months the treatment effectiveness is stable (no improvement or deterioration in PPIUS is possible) | Majority of the treatment effect of FDC tablet solifenacin 6 mg plus TOCAS observed at 3 months is already evident at 2 months | van Kerrebroeck, et al. [18] |
| Mortality is based on the UK general background mortality | The cost-effectiveness model is specific to the UK population | N/A |
| Increased mortality due to increased risk of prostate cancer is not incorporated | There are no reported data to definitively link prostate cancer to LUTS/BPH | Kopp, et al. [45]; Ørsted, et al. [46] |

BPH = benign prostatic hyperplasia; GP = general practitioner; FDC = fixed-dose combination; HS1 = Response health state; HS2 = No response health state; LUTS = lower urinary tract symptoms; TOCAS = oral controlled absorption system (OCAS™) formulation of tamsulosin; TURP = transurethral resection of the prostate.

## Supplementary Table 2. Alternative utility weights per health state

| Health state | Utility weights: EQ-5D | Utility weights: OAB-q |
| --- | --- | --- |
| Baseline | 0.848 | 0.822 |
| Response | 0.887 | 0.898 |
| No response | 0.870 | 0.851 |
| Second-line treatment | 0.879 | 0.875 |
| Withdrawal | 0.848 | 0.822 |
| Discontinuation | 0.848 | 0.822 |
| Post-surgery | 0.839 | 0.850 |
| Death | 0.000 | 0.000 |
